# Supplementary material for: Demystifying DPP III Catalyzed Peptide Hydrolysis—Computational Study of the Complete Catalytic Cycle of Human DPP III Catalyzed Tynorphin Hydrolysis
Source: Int J Mol Sci. 2022 Feb 6;23(3):1858. doi: 10.3390/ijms23031858 (PMC8836397; doi:10.3390/ijms23031858)
Supplement: Supplementary file 1 [file ijms-23-01858-s001.zip › ijms-1554646-supplementary.pdf]

SUPPORTING INFORMATION

Demystifying DPP III catalysed peptide hydrolysis - computational study of the complete catalytic cycle of human DPP III catalysed tynorphin hydrolysis.

Antonija Tomić and Sanja Tomić

Content

1. Supplementary scheme 2
2. Supplementary tables 3
3. Supplementary figures 6
4. Details of QM/MM calculations 19
5. Details of systems preparation procedure and AS(MD) simulations 20
6. References 24

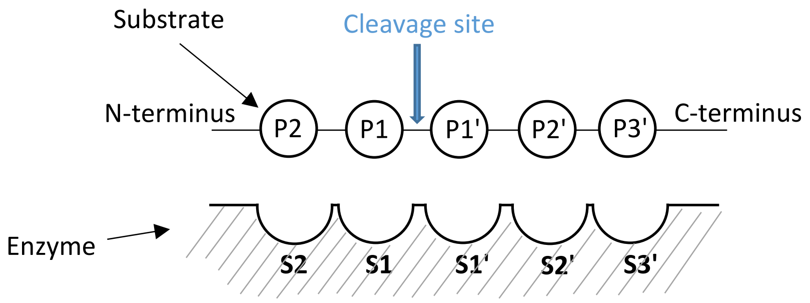

Scheme S1. Schematic representation of substrate binding to peptidase. The amino acid residues of the peptide substrate (designated as P1 to P2 and P1’ to P3’ counting from the scissile peptide bond towards the N- and C-termini of the peptides, respectively) interacts with corresponding enzyme subsites (designated as S1 to S2 and S1’ and S3’ counting from the scissile peptide bond toward the N- and C-termini of the enzyme, respectively).

Table S1. Comparison of the selected bond lengths and dihedral angles during the peptide, Leu-enkephalin, bond cleavage in the active site of human DPP III obtained within this work (shown in bold) and taken from our previous publication [30]. Calculations at the B97D/[6-31G(d) + LanL2DZ-ECP] level of theory. Atom naming as in Fig. 1.

	ES		TS1		INT1		TS2		INT2		TS3	
d(O_s-Zn) / Å	**2.45**	2.89	**2.13**	2.17	**2.10**	2.09	**2.09**	2.09	**2.09**	2.10	**2.10**	2.11
d(O_w-Zn) / Å	**2.04**	2.02	**2.18**	2.15	**2.24**	2.26	**2.18**	2.17	**2.18**	2.16	**2.18**	2.16
d(C_s-N_s) / Å	**1.34**	1.36	**1.41**	1.42	**1.42**	1.44	**1.45**	1.45	**1.52**	1.53	**1.56**	1.57
d(O_w-C_s) / Å	**2.56**	2.56	**1.69**	1.74	**1.61**	1.58	**1.51**	1.52	**1.46**	1.46	**1.44**	1.45
d(O_s-C_s) / Å	**1.26**	1.24	**1.33**	1.31	**1.34**	1.33	**1.35**	1.34	**1.35**	1.34	**1.34**	1.33
d(O_w-H_{w2}) / Å	**0.99**	0.99	**0.99**	0.99	**0.99**	0.99	**0.98**	0.98	**1.00**	1.00	**1.01**	1.01
d(O_w-H_{w1}) / Å	**1.06**	1.07	**1.57**	1.56	**1.60**	1.61	**2.11**	2.10	**2.57**	2.53	**2.49**	2.48
d(H_{w2}-O_{e2}) / Å	**1.85**	1.88	**2.65**	2.58	**2.67**	2.63	**2.49**	2.45	**1.73**	1.71	**1.63**	1.64
d(H_{w1}-O_{e1}) / Å	**1.48**	1.45	**1.03**	1.03	**1.02**	1.02	**1.00**	1.00	**1.07**	1.08	**1.21**	1.18
d(N_s-H_{w1}) / Å	**2.70**	2.78	**2.78**	2.83	**2.92**	2.80	**1.94**	1.95	**1.54**	1.53	**1.30**	1.34
d(N_s-H_{w2}) / Å	**3.90**	3.87	**3.18**	3.21	**3.14**	3.12	**2.92**	2.92	**2.52**	2.54	**2.53**	2.55
d(H450-Zn)[a] / Å	**2.13**	2.12	**2.13**	2.15	**2.13**	2.14	**2.12**	2.13	**2.12**	2.12	**2.11**	2.12
d(H455-Zn)[a] / Å	**2.13**	2.12	**2.14**	2.15	**2.14**	2.15	**2.12**	2.14	**2.12**	2.14	**2.12**	2.14
d(E508-Zn)[a] / Å	**2.08**	2.04	**2.09**	2.08	**2.09**	2.08	**2.08**	2.07	**2.07**	2.07	**2.08**	2.07
d(H568[Hε]-O_s) / Å	**1.60**	1.78	**1.46**	1.60	**1.44**	1.56	**1.41**	1.50	**1.46**	1.55	**1.49**	1.57
d(H568[Nε-Hε]) / Å	**1.06**	1.04	**1.11**	1.07	**1.12**	1.08	**1.13**	1.10	**1.11**	1.09	**1.10**	1.08
d(Y318-sub)[b] / Å	**2.29**	2.30	**2.25**	2.29	**2.26**	2.30	**2.29**	2.27	**2.05**	2.15	**2.10**	2.15
ω_1 (C-N_s-C_s-H_s)[c] / °	**−161.9**	−151.5	**−134.9**	−132.0	**−132.1**	−128.3	**158.0**	158.1	**130.2**	130.1	**127.6**	127.9
ω_2 (C'-C_s-N_s-O_s)[c] / °	**175.3**	173.7	**136.0**	138.6	**132.2**	131.2	**131.5**	132.0	**129.1**	129.8	**127.9**	128.9

	INT3		TS4		INT4	TS5	EP	EP*
d(O_s-Zn) / Å	**2.12**	2.15	**2.13**	2.13	**2.15**	**2.24**	**3.46**	2.85
d(O_w-Zn) / Å	**2.17**	2.14	**2.15**	2.15	**2.19**	**2.16**	**1.98**	2.03
d(C_s-N_s) / Å	**1.64**	1.69	**1.71**	1.74	**2.30**	**2.92**	**3.26**	3.18
d(O_w-C_s) / Å	**1.42**	1.41	**1.39**	1.39	**1.30**	**1.27**	**1.28**	1.31
d(O_s-C_s) / Å	**1.33**	1.32	**1.33**	1.31	**1.29**	**1.28**	**1.26**	1.24
d(O_w-H_{w2}) / Å	**1.05**	1.07	**1.18**	1.14	**1.61**	**2.15**	**3.15**	1.69
d(O_w-H_{w1}) / Å	**2.41**	2.41	**2.40**	2.42	**2.65**	**3.82**	**4.25**	3.58
d(H_{w2}-O_{e2}) / Å	**1.50**	1.45	**1.27**	1.31	**1.02**	**0.99**	**1.05**	1.01
d(H_{w1}-O_{e1}) / Å	**1.51**	1.57	**1.62**	1.64	**1.91**	**2.11**	**2.89**	3.56
d(N_s-H_{w1}) / Å	**1.10**	1.08	**1.06**	1.06	**1.02**	**1.02**	**1.02**	1.03
d(N_s-H_{w2}) / Å	**2.60**	2.64	**2.72**	2.72	**3.23**	**2.19**	**1.63**	4.46
d(H450-Zn)[a] / Å	**2.11**	2.11	**2.11**	2.12	**2.09**	**2.09**	**2.10**	2.10
d(H455-Zn)[a] / Å	**2.12**	2.13	**2.12**	2.14	**2.13**	**2.12**	**2.14**	2.14
d(E508-Zn)[a] / Å	**2.08**	2.07	**2.08**	2.08	**2.08**	**2.07**	**2.07**	2.04
d(H568[Hε]-O_s) / Å	**1.53**	1.61	**1.52**	1.61	**1.57**	**1.61**	**1.62**	1.81
d(H568[Nε-Hε]) / Å	**1.08**	1.07	**1.09**	1.07	**1.07**	**1.06**	**1.06**	1.04
d(Y318-sub)[b] / Å	**2.13**	2.20	**2.13**	2.21	**2.36**	**2.42**	**2.58**	2.47
ω_1 (C-N_s-C_s-H_s)[c] / °	**128.1**	128.0	**128.6**	127.9	**-**	**-**	**-**	-
ω_2 (C'-C_s-N_s-O_s)[c] / °	**126.1**	126.4	**123.7**	125.0	**-**	**-**	**-**	-

[a] Distances between the zinc ion and either nitrogen (Nδ) or oxygen (carboxyl) atoms from the histidine of glutamate amino acid residues, respectively.
[b] Distance between the oxygen atom from the Tyr318 hydroxyl group and amide hydrogen atom from the second amino acid residue from the substrate (sub) N terminus.
[c] C and C' are carbon atoms adjacent to N_s or C_s atom, respectively, while H_s is a hydrogen atom bonded to N_s.

Table S2. The energy profiles for substrate (tynorphin and Leu-enkephalin) hydrolysis in the hydrated enzyme – substrate complex system. Calculations at: (A) B97D/[6-31G(d)+LanL2DZ-ECP] and (B) B97D/[6-31G(d)+LanL2DZ-ECP] + $ZPVE_{B97D/[6-31G(d)+LanL2DZ-ECP]}$ levels of theories. Energies are in kcal/mol. Values taken from our previous publication [30] are shown in italic.

	**DPP III – tynorphin**		**DPP III – Leu-enkephalin**		***DPP III – Leu-enkephalin***	
	A	B	A	B	*A*	*B*
**ES**	0.00	0.00	0.00	0.00	*0.00*	*0.00*
**TS1**	13.50	12.41	6.95	6.39	*5.77*	*6.67*
**INT1**	10.95	10.47	6.44	6.67	*5.52*	*7.23*
**TS2**	11.82	9.25	13.13	12.11	*14.75*	*14.21*
**INT2**	0.56	1.78	9.38	8.99	*10.67*	*10.56*
**TS3**	7.19	8.23	9.81	7.76	*10.86*	*9.17*
**INT3**	5.08	5.26	8.91	8.45	*9.04*	*8.47*
**TS4**	8.88	7.03	9.35	7.34	*9.16*	*7.36*
**INT4**	-	-	6.05	6.88	-	-
**TS5**	-	-	10.98	12.54	-	-
**EP**	-0.03	-0.19	-1.04	0.22	-	-
**EP***	-	-	-	-	*-3.01*	*0.89*

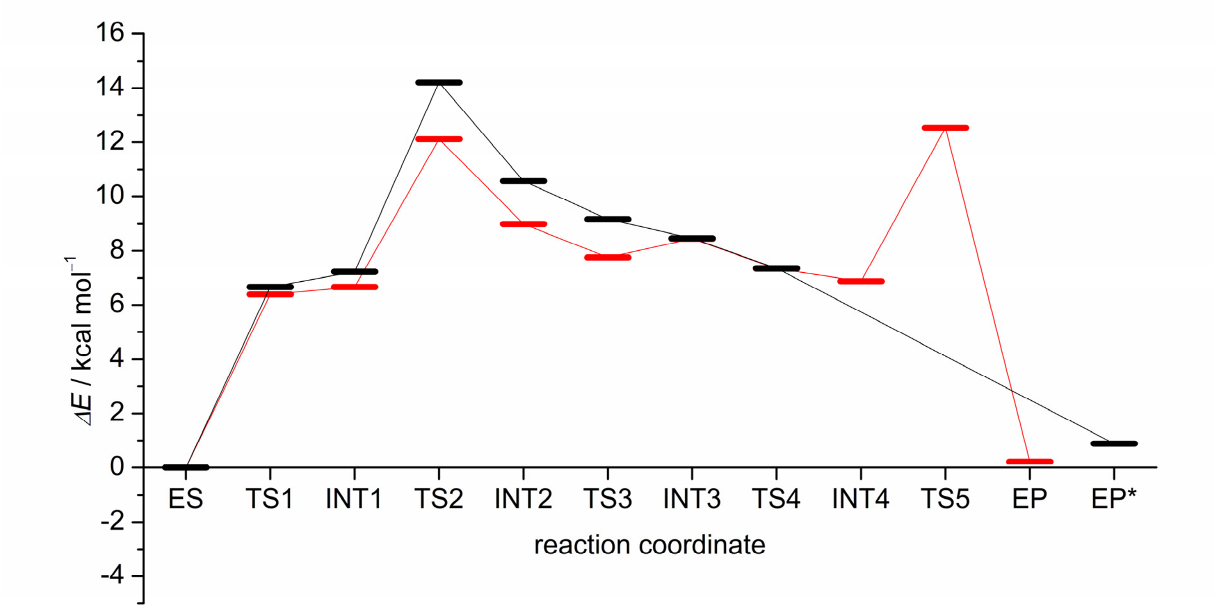

Figure S1. Comparison of energy profiles for Leu-enkephalin hydrolysis in the active site of human DPP III, calculated without MM water molecule bound to the amide nitrogen atom of the cleavable peptide bond (red) and obtained previously (black) [30]. Calculations were performed at B97D/[6-31G(d)+LanL2DZ-ECP] + $ZPVE_{B97D/[6-31G(d)+LanL2DZ-ECP]}$ level of theory.

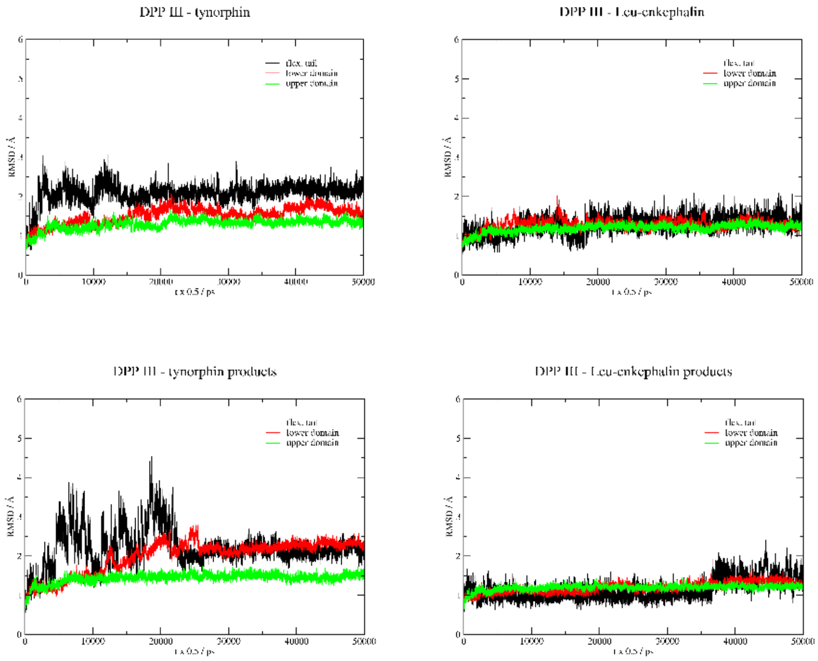

Figure S2. Enzyme backbone RMSD profile of the flexible loop (black, residues 459-492), lower (red, residues 4-335, 374-416 and 670-726) and upper (green, 336-373, 417-458 and 493-669) domain from 100 ns long conventional MD simulations.

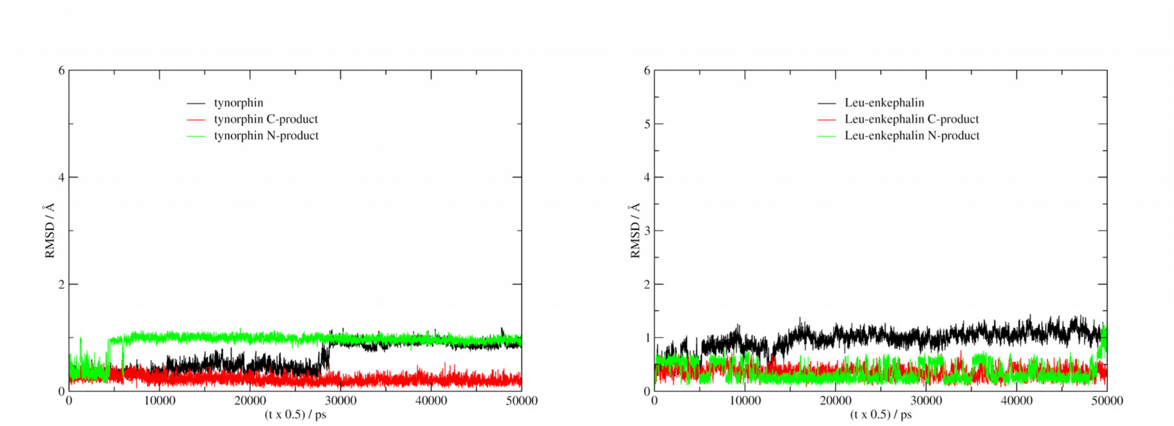

Figure S3. Substrates' (tynorphin and Leu-enkephalin) and products' backbone RMSD profiles from 100 ns long conventional MD simulations.

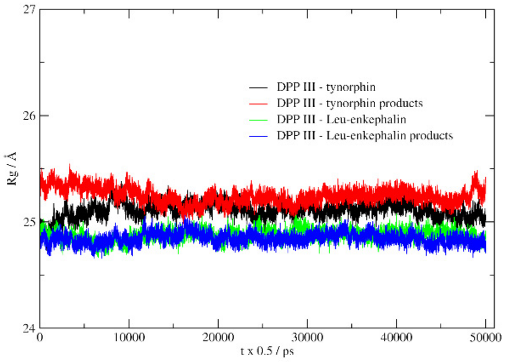

Figure S4. Radius of gyration profile of the enzyme backbone atoms (excluding residues 459-492 belonging to the protein flexible loop) obtained from 100 ns long conventional MD simulations.

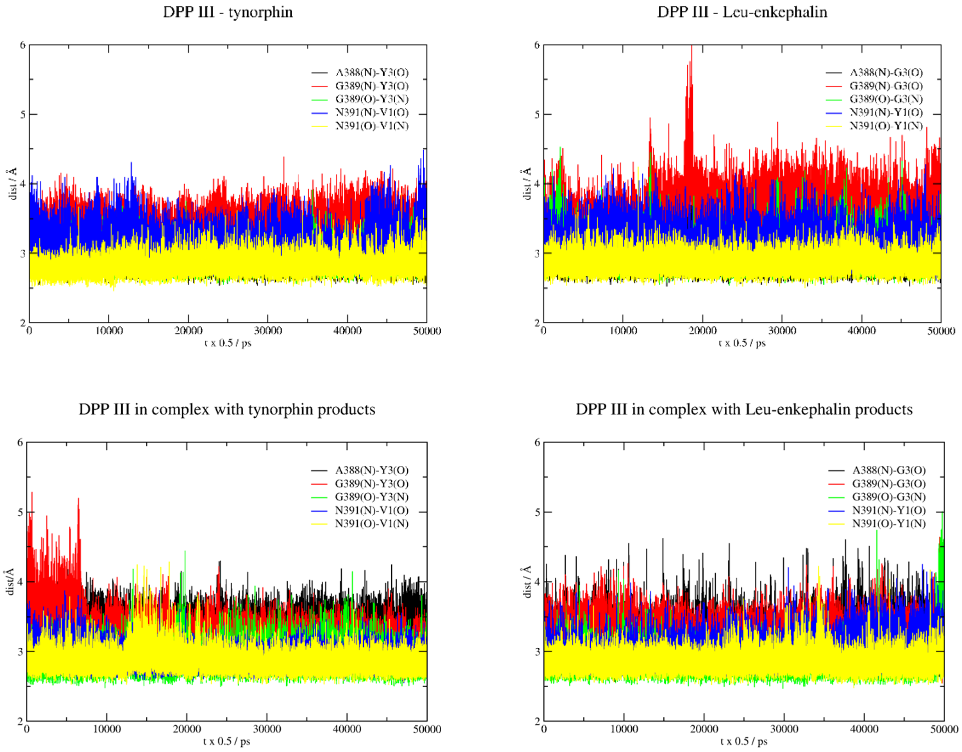

Figure S5. Selected distances between bound peptide (substrates and their C-products) and lower domain β-strand during 100 ns of conventional MD simulations.

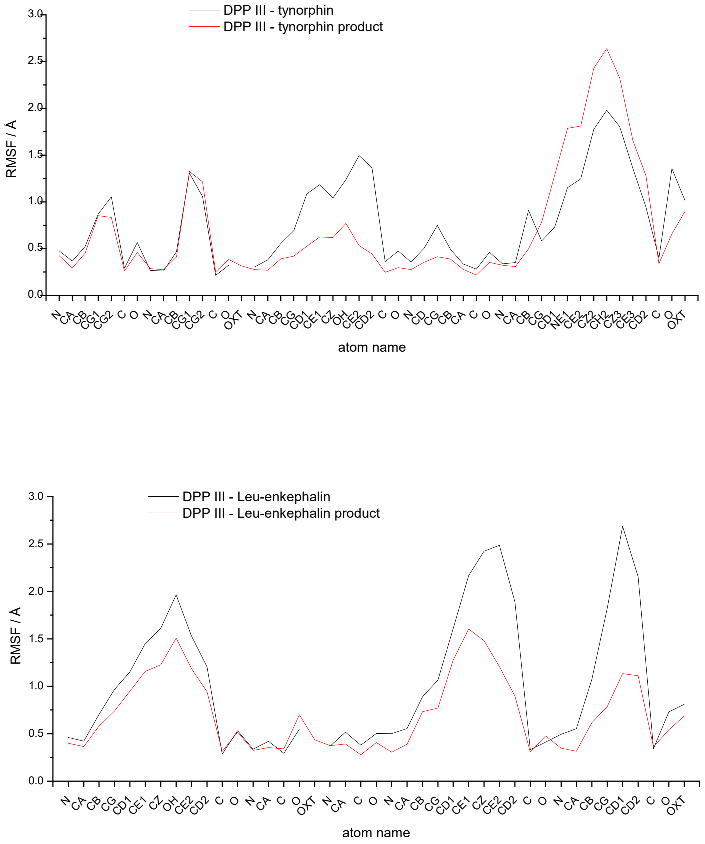

Figure S6. Substrates' (tynorphin and Leu-enkephalin) and products' heavy atoms RMSF profile obtained from 100 ns long conventional MD simulations. Prior calculations, alignment of the peptide backbone to the first structure used in the production MD simulations was performed.

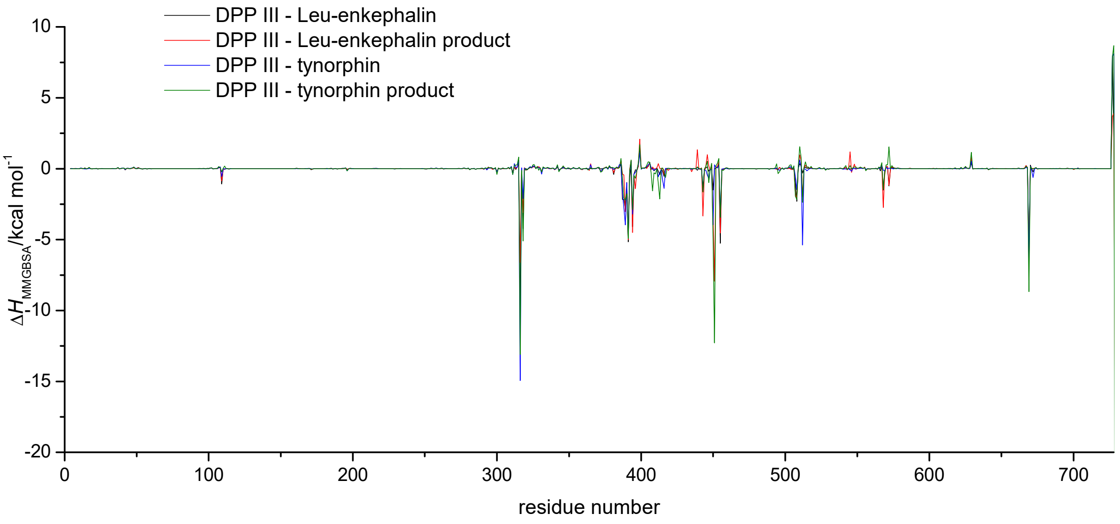

Figure S7. Per-residue binding free energies of the DPP III in complex with various ligands (substrates and their products) calculated with MM/GBSA for the last 40 ns of 100 ns long trajectories obtained by conventional MD simulations.

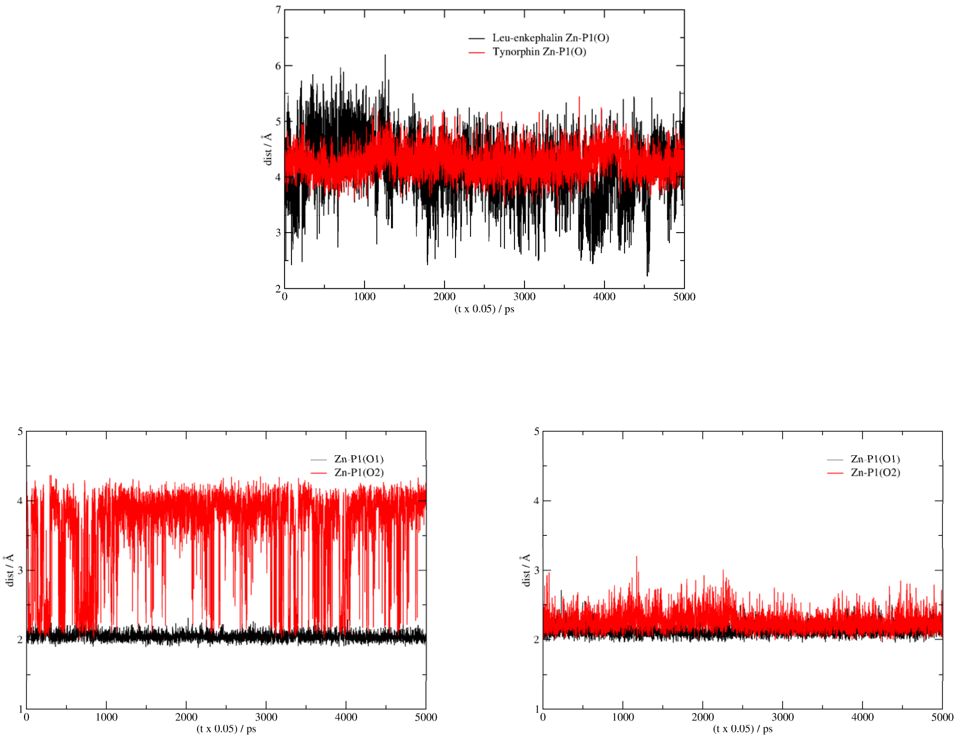

Figure S8. The substrate and C-product coordination to the zinc ion obtained from 100 ns long conventional MD simulations of DPP III in complex with: up, substrates, and down, Leu-enkephalin (left) and tynorphin (right) products. Distances to P1 carbonyl oxygen (O) atom and two oxygen atoms (O1 and O2) from the C-product carboxylate group are shown.

DPP III – Leu enkephalin

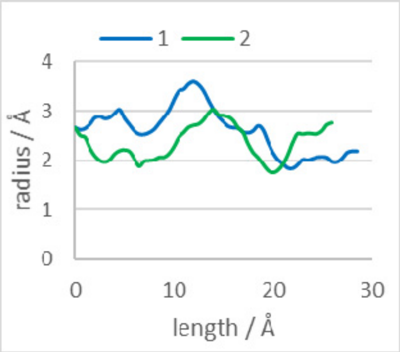

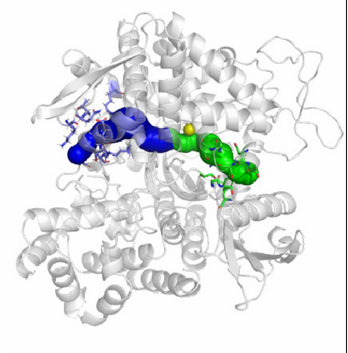

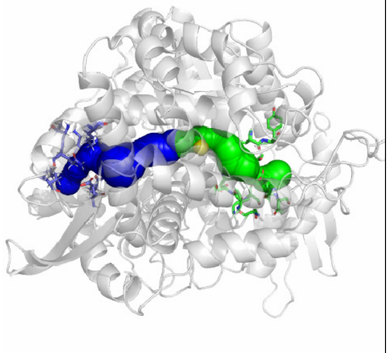

Tunnel	1 (blue)	2 (green)
bottleneck radius / Å	1.83	1.75
tunnel length / Å	27.79	25.23
throughput	0.623	0.618
curvature	1.314	1.254
Bottleneck residues	420, 670, 668, 423, 669, 667, 13, 419, 439	196, 496, 324, 317, 323, 394, 503, 504, 497, 500, 395

DPP III – Leu-enkephalin products

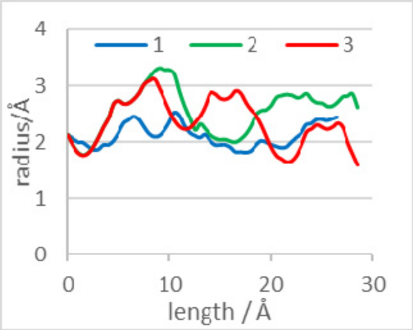

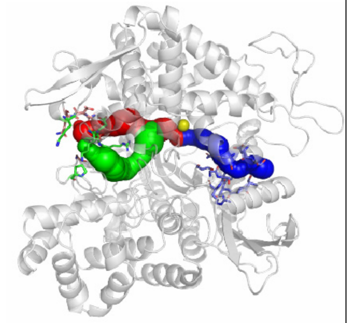

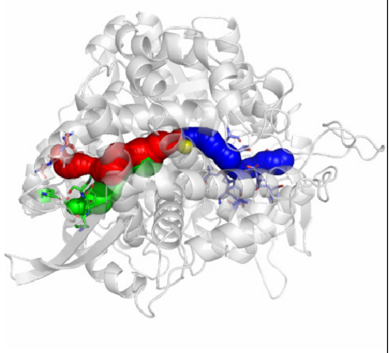

Tunnel	1 (blue)	2 (green)	3 (red)
bottleneck radius / Å	1.821	1.559	1.43
tunnel length / Å	25.991	33.687	30.40
throughput	0.559	0.520	0.497
curvature	1.249	1.411	1.310
Bottleneck residues	496, 196, 324, 195, 317, 194, 323, 325, 500, 394, 497	666, 668, 9, 667, 423, 669	419, 420, 670, 418, 13

DPP III – tynorphin

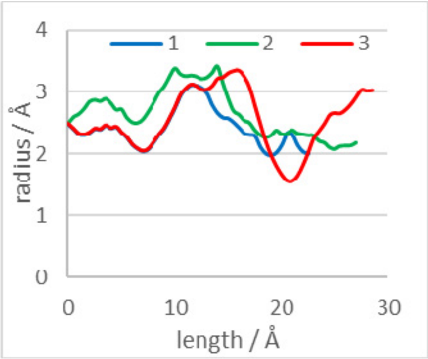

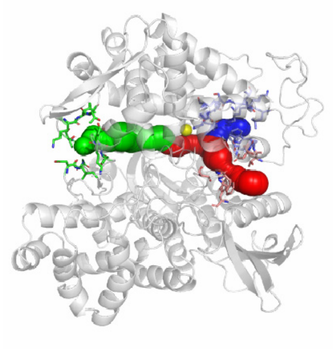

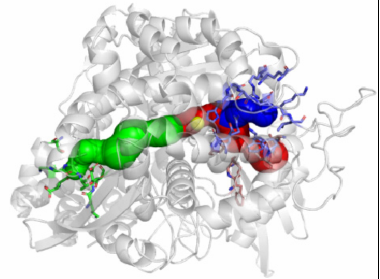

Tunnel	1 (blue)	2 (green)	3 (red)
bottleneck radius / Å	1.98	2.04	1.54
tunnel length / Å	21.71	26.30	28.05
throughput	0.682	0.651	0.622
curvature	1.173	1.225	1.268
Bottleneck residues	629, 399, 396, 458, 460, 455, 510, 457, 507, 365, 495, 459, 456	667, 423, 11, 666, 419, 668, 12, 425, 13, 424	196, 496, 323, 324, 494, 317, 322

DPP III – tynorphin products

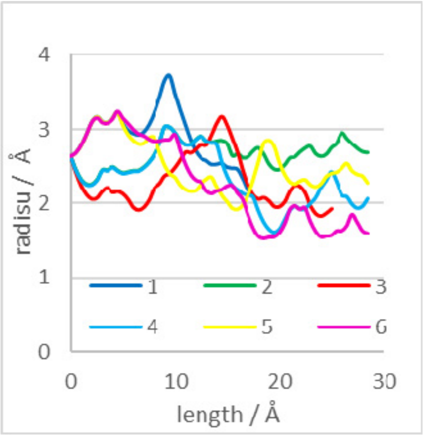

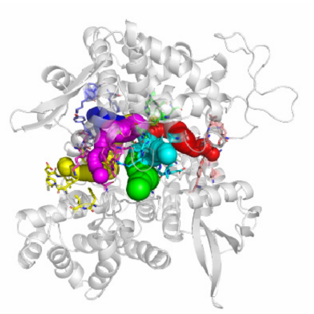

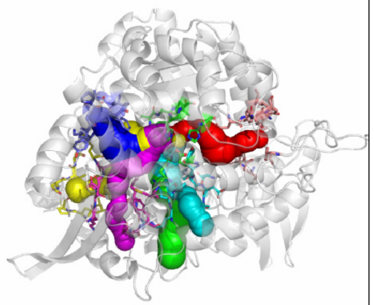

Tunnel	1 (blue)	2 (green)	3 (red)	4 (cyan)	5 (yellow)	6 (magenta)
bottleneck radius / Å	2.08	2.25	1.82	1.62	1.53	1.53
tunnel length / Å	17.16	33.55	24.47	29.94	35.58	30.82
throughput	0.768	0.640	0.605	0.568	0.512	0.468
curvature	1.266	1.293	1.239	1.530	1.616	1.303
Bottleneck residues	440, 417, 413, 439, 414, 417, 443, 415, 441, 412	568, 389, 387, 727, 388, 390, 450, 508, 318, 451, 455, 316	494, 395, 196, 294, 290, 394, 496, 396, 397	569, 318, 565, 567, 319, 321, 320, 504	668, 8, 12, 114, 13, 669, 107, 11, 9, 115, 670	109, 556, 667, 668, 107, 669, 569, 110, 666, 555

Figure S9. Tunnels calculated with the plugin CAVER 3.0 [38], implemented in the program PyMol for the equilibrated structures used as initial structures for ASMD simulations (two different views are shown, from the side and from above). Possible access routes for a probe with a minimum radius of 1.4 Å connecting the position of the carbonyl carbon atom from the scissile peptide bond and the external solvent were determined (ligand molecules were not omitted from the calculations). The zinc cation is shown as a yellow sphere. The tunnel profile is plotted as the tunnel radius versus the tunnel length. The table provides an overview of the discovered tunnels with detailed information about each tunnel.

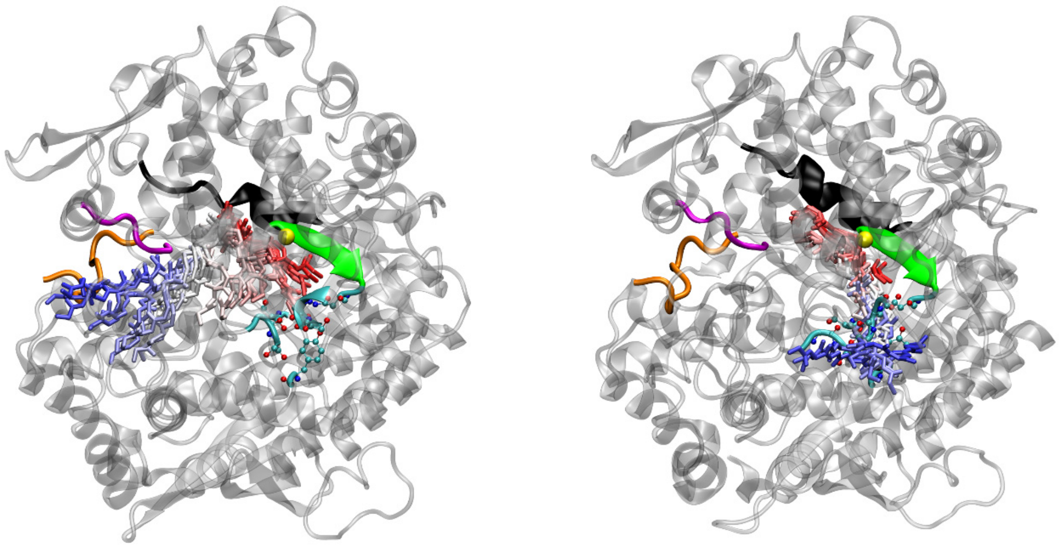

Figure S10. The exit paths of the substrates obtained from the ASMD simulations correspond to the green PMF profiles in Fig. 7. The backbone of the substrate is shown as a stick, with the atoms colored according to the evolution of the ligand exit (from red to blue). The zinc ion is shown as a yellow sphere, while the β-strand of the lower protein domain, to which the substrate is hydrogen bonded at the beginning of the simulation, is colored green. The residues forming the entrance of tunnel 1 (Y196, S317, G323, S324, N394, D496, and S497) identified by Caver are shown as a sphere and stick and colored cyan. The amino acid residues of the hinge (L409-Q420) are colored black, the loop, Lys666-Lys670 is colored magenta, and the unstructured portion of the N-terminus of the protein is colored orange.

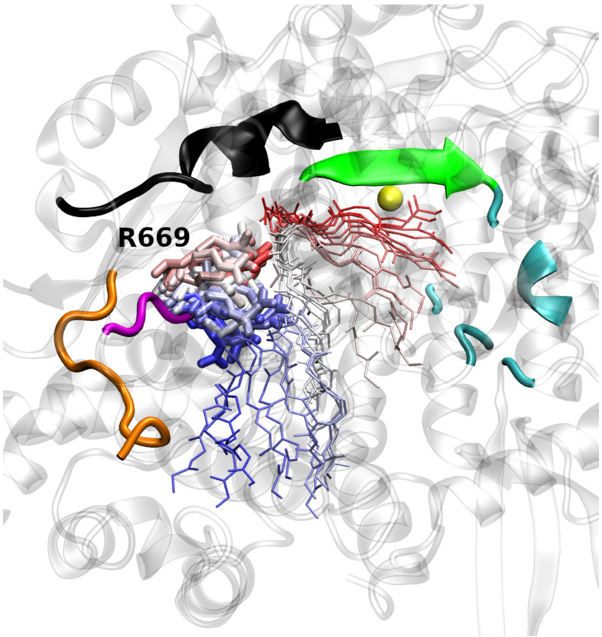

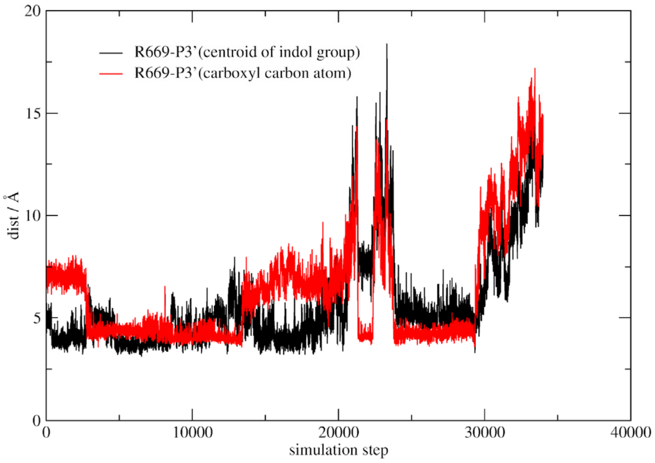

Figure S11. Top: Tynorphin output pathway from ASMD simulations corresponding to the green PMF profile in Fig. 7. Substrate backbone and R669 residues are shown in thinner and thicker sticks, respectively, with atoms colored according to the evolution of the ligand exit (from red to blue). The zinc ion is shown as a yellow sphere, while the β-strand of the lower protein domain, to which the substrate is hydrogen bonded at the beginning of the simulation, is colored green. The residues identified by Caver that form the entrance of tunnel 1 (Y196, S317, G323, S324, N394, D496, and S497) are colored cyan, the amino acid residues of the hinge (L409-Q420) are colored black, the loop residues Lys666-Lys670 are colored magenta, and the unstructured part of the N-terminus of the protein is colored orange.

Below: Distances between the centroid of the guanidino group of R669 and the centroid of the indole group of the tynorphin P3' residue (black) or the carboxylate carbon atom (red).

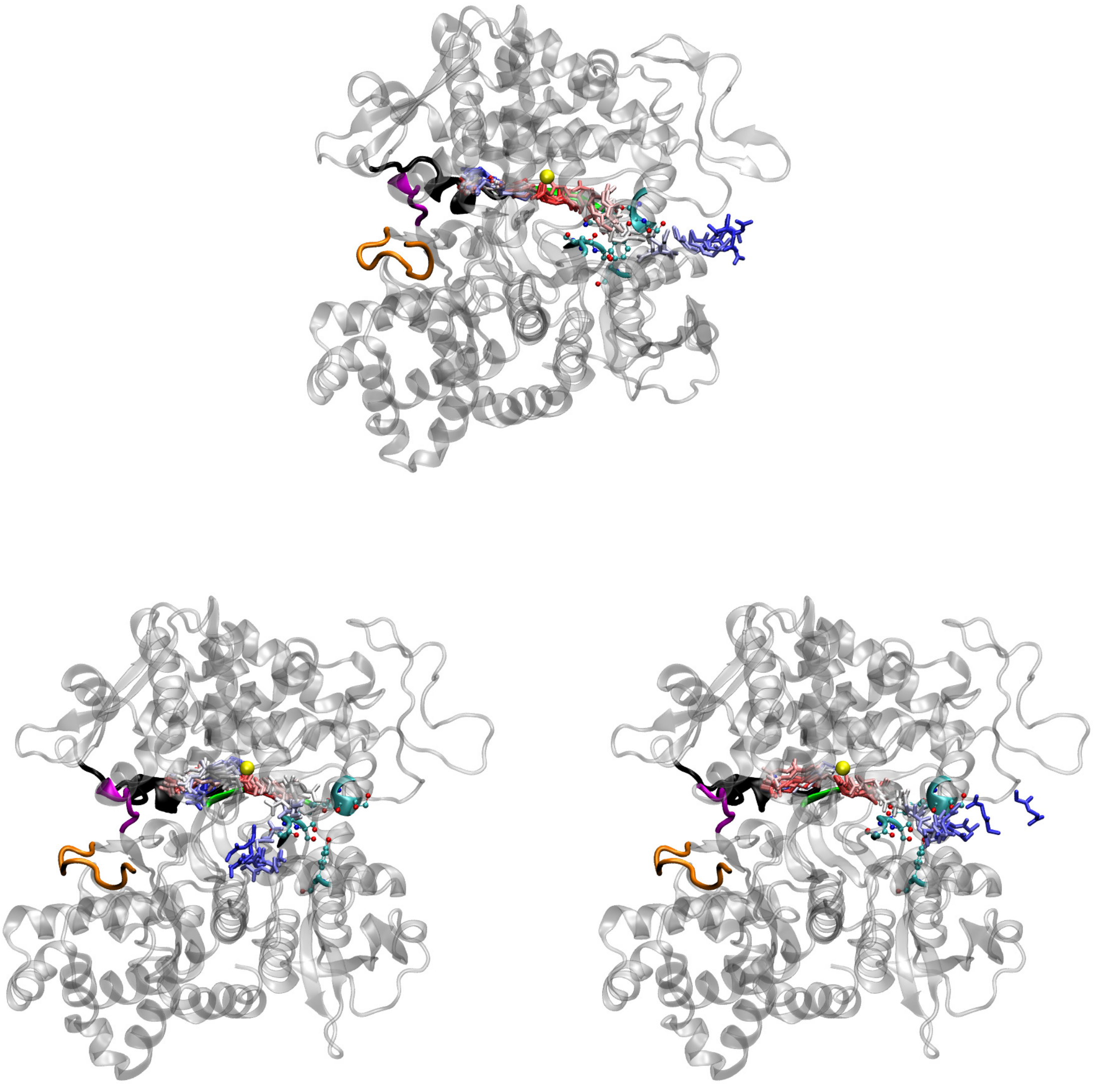

Figure S12. Leu-enkephalin (up) and tynorphin (down) C-product exit pathways from the DPP III binding site obtained from ASMD simulations, whose PMF profiles are shown in Fig. 7. For tynorphin C-product, the results of two independent ASMD simulations are shown: the black curve in Fig. 7 corresponds to the structure on the left and the blue curve in Fig. 7 corresponds to the structure on the right. The atoms of the product backbone are shown in sticks with the atoms colored according to the evolution of the ligand exit (from red to blue). The zinc ion is shown as a yellow sphere, while the β-strand of the lower protein domain, to which the substrate is hydrogen bonded at the beginning of the simulation, is colored green. The residues forming the entrance of tunnel 1 (Y196, S317, G323, S324, N394, D496, and S497) identified by Caver are colored cyan and highlighted in ball and stick representation. The amino acid residues of the hinge (L409-Q420) are colored black, the loop residues Lys666-Lys670 are colored magenta, and the unstructured portion of the N-terminus of the protein is colored orange.

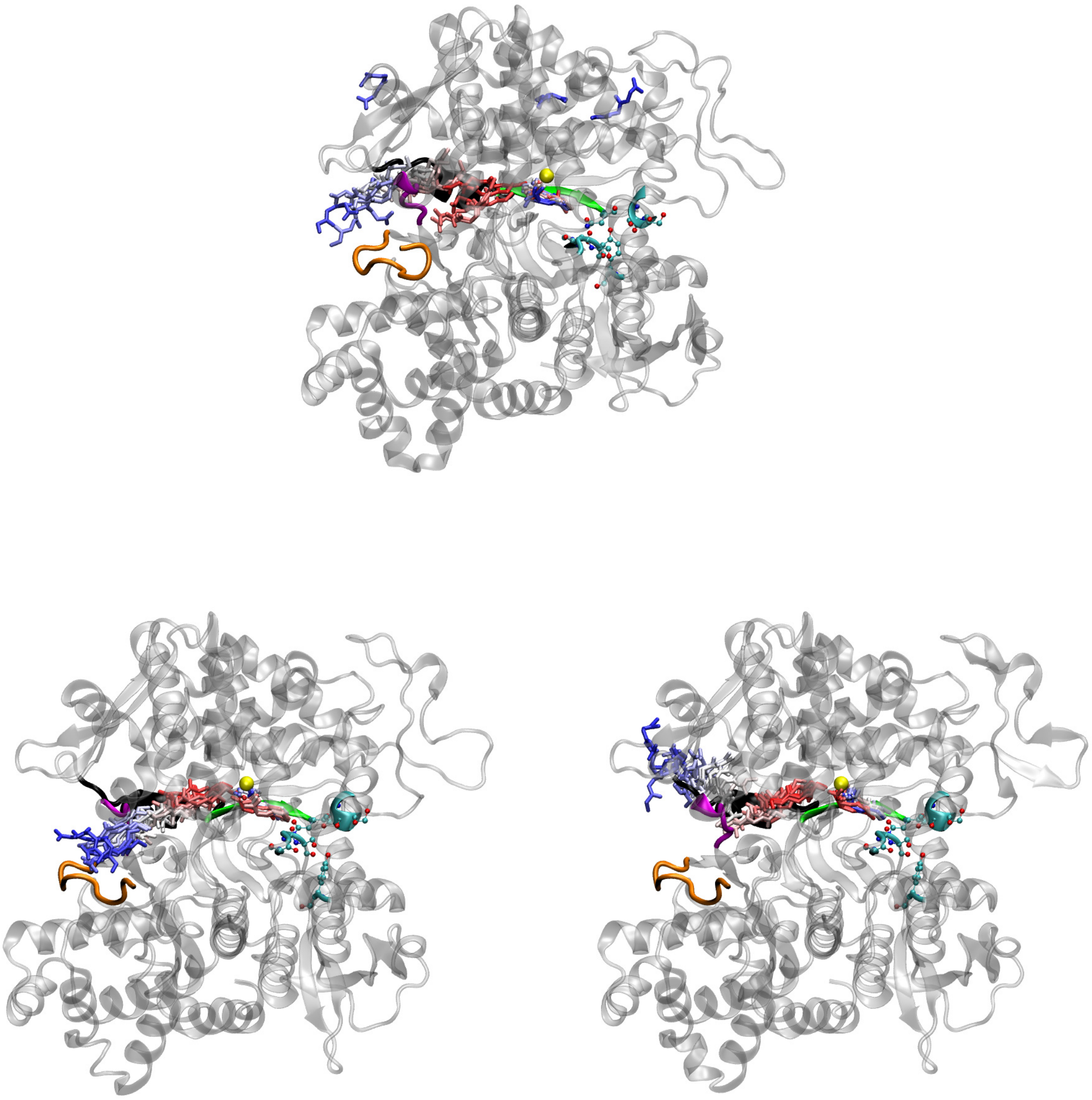

Figure S13. Leu-enkephalin (up) and tynorphin (down) N-products exit pathways from the DPP III binding site obtained from ASMD simulations, whose PMF profiles are shown in Fig. 7. The results of two independent ASMD simulations are shown for the tynorphin C-product: the red curve in Fig. 7 corresponds to the structure shown on the left and the orange curve in Fig. 7 corresponds to the structure shown on the right. The atoms of the product backbone are shown as sticks with the atoms colored according to the evolution of the ligand exit (from red to blue). The zinc ion is shown as a yellow sphere, while the β-strand of the lower protein domain, to which the substrate is hydrogen bonded at the beginning of the simulation, is colored green. The residues forming the entrance of tunnel 1 (Y196, S317, G323, S324, N394, D496, and S497) identified by Caver are colored cyan and highlighted in the sphere and bar representation. The amino acid residues of the hinge (L409-Q420) are colored black, the loop residues Lys666-Lys670 are colored magenta, and the unstructured portion of the N-terminus of the protein is colored orange.

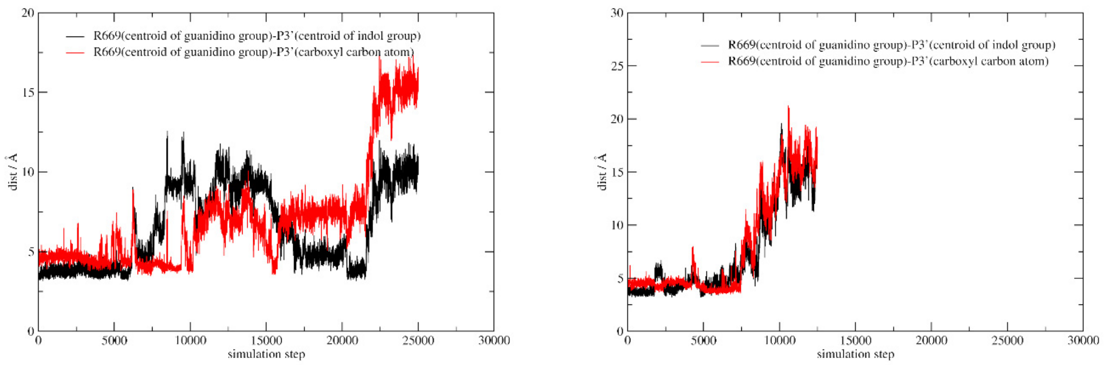

Figure S14. Distances between the centroid of the guanidino group R669 and the centroid of the indole group P3' (black) and the carboxylate carbon atom P3' (red) obtained from the ASMD simulation monitoring the release of the N-product release with the pulling speed of 0.5 Å/ns (black) and 1 Å/ns (red).

DETAILS OF QM/MM CALCULATIONS

Optimization protocol. QM/MM geometry optimization of the enzyme binding site was performed using the 2-layer ONIOM (Our own N-layered Integrated molecular Orbital and molecular Mechanics) methodology [56,57] implemented in program GAUSSIAN 09 Rev. D.01 [58]. In a two-layer ONIOM calculation the total energy of the system is obtained from three independent calculations:

$$E^{\mathrm{ONIOM}} = E^{\mathrm{real,MM}} + E^{\mathrm{model,QM}} - E^{\mathrm{model,MM}}, \quad (1)$$

The real system contains all atoms (i.e. DPP III – tynorphin and DPP III – Leu-enkephalin complexes surrounded by 3454 and 3502 water molecules, respectively) and was calculated at the molecular mechanic (MM) level of theory using the AMBER force field (parm96) [59]. The model system contains the small part of the system that is of major interest and is treated quantum mechanically (QM). Here, the side chains of Y318, H450, E451, H455, E508 and H568, the ligand (tynorphin or Leu-enkephalin), the zinc ion, and a water molecule were part of a model system and were treated using the B97D [60] DFT method and two different basis sets: 6-31G(d) for the H, N, C and O atoms, and LANL2DZ-ECP for the Zn^{2+} atom. This level of theory was used because: a) satisfactory agreement with experimental results was obtained in the Leu-enkephalin hydrolysis study [30], and b) to be able to correlate new results with the previous ones on Leu-enkephalin hydrolysis. As shown in Eq. 1, both QM and MM calculations had to be performed for the model system. Since there is a bonded interaction between the atoms in the QM and MM region, the model system contains a hydrogen link atom to saturate the open valence. The link atom was placed on the line connecting the Cα-Cβ atoms of the amino acids. Geometry optimization was performed using electronic embedding (EE) [56]. The net charge of the whole system was -23 e, while the QM region had charge +1 e. During QM/MM geometry optimization and potential energy surface scanning, protein residues and water molecules found within 8 Å of the substrate molecule were allowed to move. Minima and saddle points were determined using vibrational analysis. Transition states were confirmed by the presence of a negative eigenvalue (an imaginary frequency), with the vibrational vector leading to the desired adjacent minima. In addition, each transition state was connected to the adjacent ground states by IRC calculations.

DETAILS OF SYSTEMS PREPARATION PROCEDURE AND AS(MD) SIMULATIONS

QM/MM optimized structures of the Michaelis complex (ES) and the enzyme-product complex (EP) obtain from Leu-enkephalin and tynorphin hydrolysis study were used as a starting point in our ASMD simulations. The binding of the substrate (Leu-enkephalin and tynorphin) to the enzyme active site and the release of the products were modeled by tracking the ligand exit using ASMD simulations. For the latter, we separately simulated the release of the carboxylate part of the product (C-product) and the amino part of the product (N-product), but in the presence of the other product (N- or C-product) in the binding site.

All Arg and Lys residues in the structures were positively charged (+1e), while Glu and Asp residues were negatively charged (-1e) as expected at physiological conditions. The histidines were in a neutral state with a proton at the Nε atom, except for the histidines directly coordinating the metal ion (H450 and H455) and the histidine stabilizing the substrate during the reaction (H568), which were in a neutral state with a proton at the Nδ atom and in a protonated state (proton at the Nε and Nδ atoms), respectively, as determined in our previous publication [30]. Before performing ASMD simulations it was necessary to fully solvate the system, i.e. the complex structure (and its 1st and 2nd solvatation sphere) were placed in the truncated octahedral box filled with TIP3P water molecules (the minimum distance between any atom originally present in the solute and the edge of the periodic box was 14 Å) and Na^+ ions were added to ensure electroneutrality. For the zinc ion, the extended 4-ligand hybrid parameters for bonded/non-bonded compounds derived in our previous work were used [48].

Completely solvated system was minimized, followed heating, density equilibration and productive MD simulations. In the first cycle of optimization (1500 steps), water molecules were relaxed, while the rest of the system was harmonically restrained with a force constant of 32 kcal mol^{-1} Å^{-2}. In the second cycle (2500 steps), complex backbone and zinc ion were restrained with a force constant of 1 kcal mol^{-1} Å^{-2}. The final minimization (1500 steps) was performed without any constrains. Minimizations were performed using 470 steps of steepest descent followed by the conjugated gradient for the remaining steps. The energy optimized system was heated from 0 to 300 K and equilibrated in five stages. In the first stage, system was heated from 0 K to 300 K during 250 ps using *NVT* ensemble with an integration step of 1 fs. During this stage complex and zinc ion were constrained with 10 kcal mol^{-1} Å^{-2}. To bring the density of our system to equilibrium, we performed a 500 ps *NpT* ensamble simulation with an integration step of 2 fs and constraints of 20 mol^{-1} Å^{-2} on entire complex. In the next four stages of equilibration the restraints of the backbone atoms were gradually reduced from 10 to 0 through a series of *NVT* MD simulations with an integration step of 2 fs: 200 ps with constrains of 10 kcal mol^{-1} Å^{-2}, 200 ps with constrains of 5 kcal mol^{-1} Å^{-2}, 200 ps with constrains of 1 kcal mol^{-1} Å^{-2} and during the final 2 000 ps all constrains were released. The SHAKE algorithm was used to constrain covalent bonds involving hydrogens. The pressure was maintained with Berendsen barostat at 1 atm with the pressure relaxation time of 1 ps, while the system temperature was held constant at 300 K using the Langevin thermostat with a collision frequency of 1 ps^{-1}. Simulations were performed using periodic boundary conditions with a cutoff value of 11 Å while the particle mesh Ewald (PME) method was used for calculation of the long-range electrostatic interactions [61,62].

The initial structures for ASMD simulations were equilibrated structures of DPP III complexes with substrates and their ligands. Since DPP III is by nature a rather flexible enzyme with its binding site

located deep in a gap between the two domains, it was a major challenge to choose the correct reaction coordinate that would allow us to properly monitor the release of substrates and products. Therefore, to model the exit of the substrate and products from the enzyme binding sites different reaction coordinates were used (*vide infra*). In addition, different pulling speeds (from 5 to 0.167 Å/ns) and force constants (from 50 to 5 kcal mol-1 Å-2) were tested to study their effects on the PMF profiles and consequently to choose those that represent the best compromise between simulation velocity and conformational sampling.

Parameters were tested in ASMD simulations monitoring the release of Leu-enkephalin and its products from the DPP III binding site. The reaction coordinate was the end-to-end distance between the zinc ion and the center of mass of the backbone heavy atoms belonging to the following: a) amino acid residues from the scissile peptide bond to the N-terminus of the ligand (P2 and P1) in the case of substrate and C-product release, and b) amino acids from the scissile peptide bond to the C-terminus of the ligand (P1'-P3') in the case of N-product release. As shown in Fig. S15, a further decrease in pulling velocity from 0.5 to 0.25 Å/ns had no effect on the PMF profile obtained for Leu-enkephalin C-product release. In all simulations, the C-product left the enzyme binding site without significant opening of the protein. In the ASMD simulations monitoring the release of Leu-enkephalin, exit of the substrate from the protein binding site was accompanied by protein opening and re-closure of the protein (indicated by a decrease in protein globularity, see Fig. S16a) when pulling velocity of 2.5 Å/ns and a force constant of 25 kcal mol^{-1} $Å^{-2}$ were applied, whereas it remained bound to the β-strand of the lower domain when pulling velocity of 0.5 Å/ns and a force constant of 5 kcal mol^{-1} $Å^{-2}$ were used. Interestingly, similar behavior resulted from AMSD simulations in which we followed the release of tynorphin and its C-product from the enzyme binding site using the same reaction coordinate and different sets of parameters (Fig. S16b). Indeed, the change in reaction coordinate was mainly the result of the complete opening of the protein, while the ligand remained bound to the β-strand of the lower protein domain (Fig. S16c). Therefore, to force the exit of the ligand and its detachment from the β-strand of the lower protein domain different reaction coordinates had to be used to monitor the release of substrates and C-products.

The final sets of ASMD simulations were performed with a force constant of 5 kcal mol^{-1} $Å^{-2}$ and pulling velocity of 0.5 or 1 Å/ns wherein reaction coordinate was partitioned into 25 equal segments (each 1 Å in long) and either 25 (each 2 ns long) or 50 (each 1 ns long) trajectories were simulated per stage. After each stage (change in reaction coordinated), the structure closest to the Jarzynski average was determined and used as the starting point for the next step. The simulation parameters used during the ASMD runs were the same as those used during the final stage of equilibration. The release of substrate from the binding site of the enzyme was traced by defining the reaction coordinate as the end-to-end distance between the center of mass of the backbone heavy atoms of the peptide P2 residue and the β-strand (Ala388-Asn391 residues) of the lower protein domain. In order to successfully track the release of products from the enzyme binding site, various reaction coordinates were used, in particular the end-to-end distance between: (a) the heavy atoms of the backbone of P2 residue and the β-fold of the lower domain of the protein (Ala388-Asn391) to monitor the step of release of the C-product of tynorphin, (b) the zinc ion and the center of mass of the backbone heavy atoms of the amino acids P1 and P2 to release the Leu-enkephalin C-product, and (c) the zinc ion and the center of mass of the heavy atoms of the amino acids P1', P2' and P3' to release the N-products.

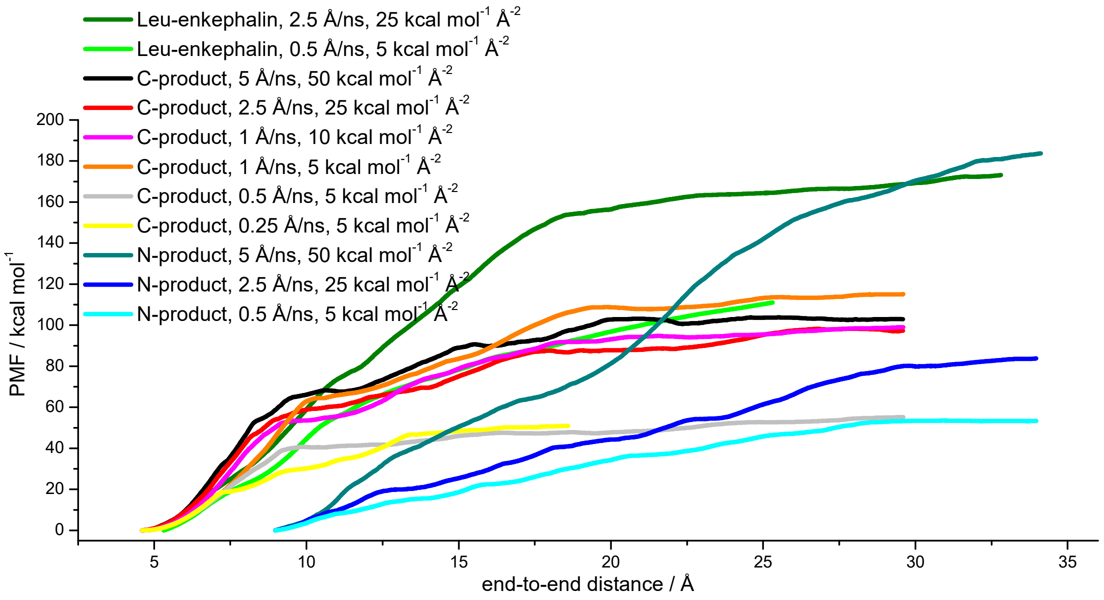

Figure S15. PMFs obtained from ASMD simulations modeling the release of the Leu-enkephalin and its products from the DPP III binding site. Different pulling velocities, *v* (in Å/ns), and force constants, *k* (in kcal mol^{-1} Å^{-2}), were tested. The reaction coordinate is the end-to-end distance between the zinc ion and the center of mass of the backbone heavy atoms belonging to: a) amino acid residues from the scissile peptide bond to the N-terminus of the ligand (P2 and P1) in the case of substrate and C-product release, and b) amino acids from the scissile peptide bond to the C-terminus of the ligand (P1'-P3') in the case of N-product release. Reaction coordinate was partitioned into 25 equal segments (1 Å in length) and 25 trajectories (either 1, 2 or 4 ns long) were simulated per each, except simulations monitoring C- and N-product release with v=5 Å/ns and k=50 kcal mol^{-1} Å^{-2} where the reaction coordinate was divided into 5 (5 Å in length) segments, and Leu-enkephalin, C- and N-product release with v=2.5 Å/ns and k=25 kcal mol^{-1} Å^{-2} where reaction coordinate was divided into 10 (2.5 Å in length) segments.

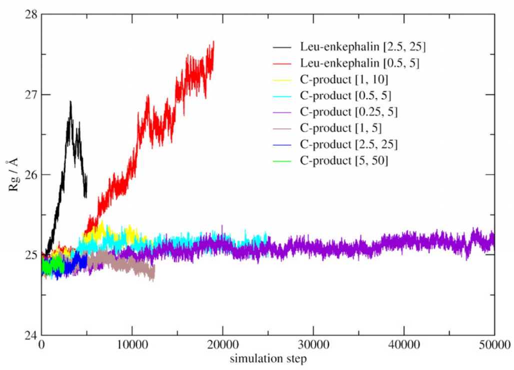

a)

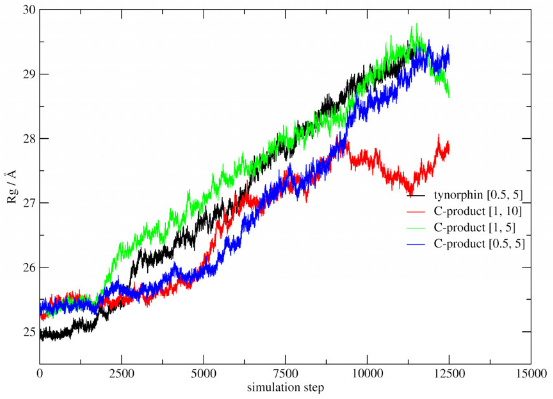

b)

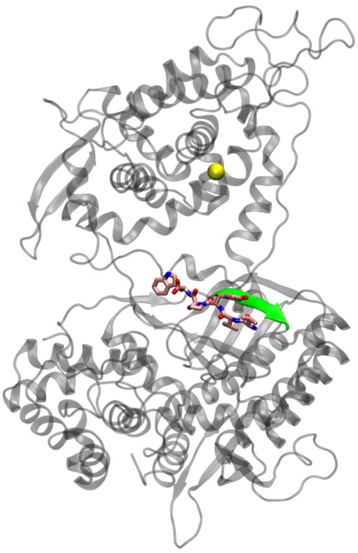

c)

Figure S16. Radius of gyration profiles of the protein backbone atoms (excluding residues belonging to the protein flexible loop 463-489) calculated from the trajectories determined to be closest to the Jarzynski average. Trajectories are obtained from ASMD simulations of DPP III in complex with: a) Leu-enkephalin (and its C-product) and b) tynorphin (and its C-product). In square brackets values of pulling velocity (in Å/ns) and force constant (in kcal mol^{-1} Å^{-2}) applied in ASMD simulations are listed. c) Structure of the DPP III – tynorphin complex obtained at the end of ASMD simulation with pulling velocity of 0.5 Å/ns and force constant of 5 kcal mol^{-1} Å^{-2}. Zinc ion is shown as yellow sphere, tynorphin as sticks with carbon atoms colored pink (hydrogens are not shown) and the β-strand from the lower protein domain (Ala388-Asn391 residues) is colored green.

REFERENCES:

[56] Vreven, T.; Morokuma, K.; Farkas, Ö.; Schlegel, H.B.; Frisch, M.J. Geometry optimization with QM/MM, ONIOM, and other combined methods. I. Microiterations and constraints. *J. Comput. Chem.* **2003**, *24*, 760–769, doi:10.1002/jcc.10156.

[57] Dapprich, S.; Komáromi, I.; Byun, K.S.; Morokuma, K.; Frisch, M.J. A new ONIOM implementation in Gaussian98. Part I. The calculation of energies, gradients, vibrational frequencies and electric field derivatives. *J. Mol. Struct. THEOCHEM* **1999**, *461–462*, 1–21, doi:10.1016/S0166-1280(98)00475-8.

[58] M. J. Frisch, G. W. Trucks, H. B. Schlegel, G. E. Scuseria, M. A. Robb, J. R. Cheeseman, G. Scalmani, V. Barone, B. Mennucci, G. A. Petersson, H. Nakatsuji, M. Caricato, X. Li, H. P. Hratchian, A. F. Izmaylov, J. Bloino, G. Zheng, J. L. Sonnenberg, M. Hada, M. Ehara, K. Toyota, R. Fukuda, J. Hasegawa, M. Ishida, T. Nakajima, Y. Honda, O. Kitao, H. Nakai, T. Vreven, J. A. Montgomery, Jr., J. E. Peralta, F. Ogliaro, M. Bearpark, J. J. Heyd, E. Brothers, K. N. Kudin, V. N. Staroverov, R. Kobayashi, J. Normand, K. Raghavachari, A. Rendell, J. C. Burant, S. S. Iyengar, J. Tomasi, M. Cossi, N. Rega, J. M. Millam, M. Klene, J. E. Knox, J. B. Cross, V. Bakken, C. Adamo, J. Jaramillo, R. Gomperts, R. E. Stratmann, O. Yazyev, A. J. Austin, R. Cammi, C. Pomelli, J. W. Ochterski, R. L. Martin, K. Morokuma, V. G. Zakrzewski, G. A. Voth, P. Salvador, J. J. Dannenberg, S. Dapprich, A. D. Daniels, O¨. Farkas, J. B. Foresman, J. V. Ortiz, J. Cioslowski and D. J. Fox, Gaussian 09, Revision D.01, Gaussian, Inc., Wallingford, CT, 2009.

[59] Cornell, W.D.; Cieplak, P.; Bayly, C.I.; Gould, I.R.; Merz, K.M.; Ferguson, D.M.; Spellmeyer, D.C.; Fox, T.; Caldwell, J.W.; Kollman, P.A. A Second Generation Force Field for the Simulation of Proteins, Nucleic Acids, and Organic Molecules. *J. Am. Chem. Soc.* **1995**, *117*, 5179–5197, doi:10.1021/ja00124a002.

[60] Grimme, S. Semiempirical GGA-type density functional constructed with a long-range dispersion correction. *J. Comput. Chem.* **2006**, *27*, 1787–1799, doi:10.1002/jcc.20495.

[61] Darden, T.; York, D.; Pedersen, L. Particle mesh Ewald: An N·log(N) method for Ewald sums in large systems. *J. Chem. Phys.* **1993**, *98*, 10089, doi:10.1063/1.464397.

[62] Essmann, U.; Perera, L.; Berkowitz, M.L.; Darden, T.; Lee, H.; Pedersen, L.G. A smooth particle mesh Ewald method. *J. Chem. Phys.* **1995**, *103*, 8577–8593, doi:10.1063/1.470117.
